# Implementation of a common data model in Health Data Research Network Canada: Lessons learned

**DOI:** 10.23889/ijpds.v9i5.2833

**Published:** 2024-09-18

**Authors:** Lisa Lix, Georgina Archbold, HDRN Canada CDM Working Group, Mahmoud Azimzee

**Affiliations:** 1 University of Manitoba; 2 Health Data Research Network Canada; 3 ICES

## Background

The Observational Medical Outcomes Partnership (OMOP) Common Data Model (CDM) is a standardized structure for observational data that enables collaborative health research. Health Data Research Network (HDRN Canada) is conducting a pilot study to implement this standard at partner data linkage centers.

## Objective

Our objective is to describe the process used to implement the OMOP CDM, methodological and technical challenges, and the approach to evaluate CDM implementation at multiple sites.

## Approach

CDM working group members include technical staff from data linkage centers in four provinces and a national data coordinating center, HDRN Canada operational and executive leads, and OMOP CDM consultants. The evaluation aims to collect quantitative and qualitative information on Extract, Transform, and Load (ETL) processes, performance of the CDM in a multi-site observational case study, and data quality.

## Results

Initial steps have included collaborative learning about the CDM structure, importing open-source tools and licensed coding standards (i.e., Systematized Nomenclature of Medicine-Clinical Terms [SNOMED-CT], RxNorm) at data linkage centres, and exploring the technical requirements for developing ETL processes and hosting OMOP data. Creation of select OMOP tables has been accompanied by discussions about models for mapping clinical and prescription drug coding standards. A scoping review of evaluation strategies is underway.

## Conclusions and Implications

The benefits of standardizing health data for multi-site research and supporting the production of findable, accessible, interoperable and reusable (FAIR) national data underscores the importance of HDRN Canada embarking on this OMOP CDM implementation project. The lessons learned will benefit other multi-site data standardization initiatives.

